# Acupuncture for ankle sprain: systematic review and meta-analysis

**DOI:** 10.1186/1472-6882-13-55

**Published:** 2013-03-04

**Authors:** Jimin Park, Seokyung Hahn, Ji-Yeun Park, Hi-Joon Park, Hyangsook Lee

**Affiliations:** 1 Department of Acupuncture and Moxibustion, College of Korean Medicine, Kyung Hee University, Kyung Hee Dae-ro 26, Dongdaemun-gu, Seoul, 130-701, Korea; 2 Department of Medicine, Seoul National University College of Medicine, Seoul, Korea; 3 Department of Korean Medical Science, Graduate School, Kyung Hee University, Seoul, Korea; 4 Acupuncture and Meridian Science Research Center, College of Korean Medicine, Kyung Hee University, Seoul, Korea

**Keywords:** Acupuncture, Ankle sprain, Systematic review, Randomized controlled trial, Meta-analysis

## Abstract

**Background:**

Ankle sprain is one of the most frequently encountered musculoskeletal injuries; however, the efficacy of acupuncture in treating ankle sprains remains uncertain. We therefore performed a systematic review to evaluate the evidence regarding acupuncture for ankle sprains.

**Methods:**

We searched 15 data sources and two trial registries up to February 2012. Randomized controlled trials of acupuncture were included if they involved patients with ankle sprains and reported outcomes of symptom improvement, including pain. A Cochrane risk of bias assessment tool was used. Risk ratio (RR) or mean difference (MD) was calculated with 95% confidence intervals (CIs) in a random effects model. Subgroup analyses were performed based on acupuncture type, grade of sprain, and control type. Sensitivity analyses were also performed with respect to risk of bias, sample size, and outcomes reported.

**Results:**

Seventeen trials involving 1820 participants were included. Trial quality was generally poor, with just three reporting adequate methods of randomization and only one a method of allocation concealment. Significantly more participants in acupuncture groups reported global symptom improvement compared with no acupuncture groups (RR of symptoms persisting with acupuncture = 0.56, 95% CI 0.42–0.77). However, this is probably an overestimate due to the heterogeneity (*I*^*2*^ = 51%) and high risk of bias of the included studies. Acupuncture as an add-on treatment also improved global symptoms compared with other treatments only, without significant variability (RR 0.61, 95% CI 0.51–0.73, *I*^2^ = 1%). The benefit of acupuncture remained significant when the analysis was limited to two studies with a low risk of bias. Acupuncture was more effective than various controls in relieving pain, facilitating return to normal activity, and promoting quality of life, but these analyses were based on only a small number of studies. Acupuncture did not appear to be associated with adverse events.

**Conclusions:**

Given methodological shortcomings and the small number of high-quality primary studies, the available evidence is insufficient to recommend acupuncture as an evidence-based treatment option. This calls for further rigorous investigations.

## Background

Acute ankle sprain is an acute injury of one or more of the ankle ligaments. Among the tendon and ligament injuries presenting to physicians, acute ankle sprain is one of the most commonly encountered musculoskeletal injuries in both athletes and sedentary people. Ankle sprains result in high costs to society due to increased healthcare resource use and work absence. It has been estimated that ankle sprain occurs at a rate of one injury per 10,000 people every day in the US, accounting for an estimated 2 million injuries per year and 20% of all sports injuries [1,2].

Ankle sprains are classified into three grades depending on the severity of the injury: grade I is mild stretching or partial tear of the anterior talofibular and/or calcaneofibular ligaments accompanied by mild tenderness and swelling but with slight or no functional loss; grade II is incomplete tear of ligaments with moderate pain, swelling, and functional loss; and grade III is characterized by complete tear of ligaments that results in severe swelling, pain, and loss of function and motion [3].

The main goals of treatment are to relieve pain, maintain range of motion (ROM), return to pre-injury level, and prevent recurrence of injury. Among many different treatment options used for ankle sprains, the three major types of treatment are conservative, functional, and surgical. Conservative treatment means plaster cast immobilization and functional treatment indicates early mobilization using external supports (e.g. elastic bandage, tape, orthotic support) plus coordination training [4]. For patients with grade I or II injury, early use of PRICE (protection, rest, ice, compression, and elevation), ankle support, and maintaining ROM are necessary. For patients with grade III injury, surgical treatment is recommended [3]. In addition to these treatments, analgesics such as acetaminophen and non-steroidal anti-inflammatory drugs (NSAIDs) are commonly used as an adjunct. Therapeutic ultrasonography and short-wave diathermy are also commonly used, but there is little evidence to promote their use in terms of symptom relief [5,6].

In addition to conventional treatments for ankle sprains, complementary and alternative therapies such as herbs and homeopathy have been thought to relieve pain, reduce swelling, and help the body restore damaged tissue, but the evidence is limited [7,8]. Acupuncture, one of the most commonly used therapeutic modalities in complementary and alternative medicine, is used extensively for painful conditions [9,10]. One survey reported that 76% of responding American physicians used acupuncture for ankle sprain and 90% of them assessed its efficacy as very/somewhat effective [11]. In 2009, approximately 2.8 million Korean people were diagnosed with an ankle injury, making ankle injury the fifth most common reason for visits to Korean Medicine clinics, and of them 1.2 million sought acupuncture treatment [12]. Clinical experience and some animal studies indicate that ankle sprain responds rapidly to acupuncture, which alleviates the intensity and duration of pain, contributing to a prompt return to pre-injury activity [13,14]. Given its popular use and claimed effectiveness, however, the evidence in support of acupuncture for treating ankle sprain remains unclear. Because there is no convincing information on the efficacy of acupuncture for ankle sprains, we decided to critically evaluate the evidence.

## Methods

### Eligibility criteria

#### Types of study

All randomized controlled trials (RCTs) evaluating acupuncture treatment for ankle sprains were considered.

#### Types of participant

Studies enrolling patients who reported an ankle sprain regardless of duration were eligible for inclusion. The diagnosis could be based on any method, including physical examination (positive anterior drawer test, pain, and swelling), arthrography, or a stress radiograph of the injured ankle. Trials including patients with congenital deformities, degenerative conditions, or fractures were excluded. Mixed population studies including adults and children were included.

#### Types of intervention

Acupuncture included needle acupuncture, ear acupuncture, electroacupuncture, pharmacopuncture (injection of herbal medicine into acupuncture points), bee venom acupuncture, scalp acupuncture, warm acupuncture, and moxibustion. Studies that assessed the combined effect of acupuncture plus other related treatments (e.g. acupuncture plus moxibustion) were also considered. We did not include trials testing non-penetrating acupuncture point stimulation (e.g. acupressure, transcutaneous electrical nerve stimulation (TENS), magnets). Trials comparing different forms of acupuncture were excluded because the efficacy of the control intervention could not be determined. Details of acupuncture interventions were extracted and tabulated based on the revised Standards for Reporting Interventions in Clinical Trials of Acupuncture (STRICTA) [15].

#### Types of control

For control groups, we considered placebo, usual care, and no intervention. Sham or placebo acupuncture intervention means use of a non-penetrating sham needle or superficial needling at non-acupuncture points. Usual care includes PRICE, analgesic drugs, functional exercise, and/or electrotherapy such as ultrasound or short waves. When acupuncture was given with other usual treatment, we included only those trials where identical usual treatment was administered to the acupuncture and control groups.

#### Types of outcome measure

The primary outcome of this systematic review was patient-reported global symptom improvement at the end of treatment. Pain intensity data were included in the review if data for global symptoms were not provided.

Secondary outcomes included time to achieve pre-injury level of work or sports, subjective (e.g. giving way) and objective (e.g. inversion stress test, talar tilt, anterior drawer test, postural sway analysis) evaluations of ankle instability, dichotomous (e.g. yes or no) and continuous (e.g. visual analog scale (VAS)) data regarding swelling, recurrence of ankle sprain, subsequent surgery, or long-term treatment, health-related quality of life (e.g. Short Form 36 (SF-36)), and adverse events related to acupuncture treatment.

### Literature search

We searched the following databases from their inception to February 2012; Cochrane Central Register of Controlled Trials, PubMed, Ovid EMBASE, the Cumulative Index to Nursing and Allied Health Literature (CINAHL), SPORTDiscus, the Allied and Complementary Medicine Database (AMED), Rehabilitation and Sports Medicine Source, and the China National Knowledge Infrastructure databases (CNKI). We also searched Korean databases including the Oriental Medicine Advanced Searching Integrated System, the Korean Studies Information Service System, RISS4U, Korea Institute of Science and Technology Information, KOREAMED, DBPIA, and the Korea National Assembly Library. Ongoing trials were searched in trial registries at http://www.controlled-trials.com and http://www.clinicaltrials.gov. The reference lists of reviews and relevant articles were screened for additional studies.

Search terms used for the Cochrane Central Register of Controlled Trials were as follows: (“ankle injuries”[MeSH] OR “sprains and strains”[MeSH] OR “sprain*”[ti, ab, kw] OR “strain*”[ti, ab, kw] OR “injur*”[ti, ab, kw] OR “ankle*”[ti, ab, kw]) AND (“acupuncture”[MeSH] OR “acupuncture therapy”[MeSH] OR “acupunc*”[ti, ab, kw] OR “electroacupunc*”[ti, ab, kw] OR “meridian*”[ti, ab, kw] OR “acupoint*”[ti, ab, kw] OR “moxibustion*”[ti, ab, kw] OR “moxa*”[ti, ab, kw]). These search terms were slightly modified for other databases. Trials published in English, Korean, or Chinese were sought.

### Study selection and data extraction

Two reviewers (Jimin Park and Ji-Yeun Park) independently reviewed all searched articles to evaluate their suitability for inclusion. If there was disagreement, it was resolved by discussion between the reviewers; further information was sought from the original authors if necessary.

After selection of studies, the aforementioned two reviewers independently extracted the following data from the selected articles: author, year of publication, country, study design, participants (age, gender), duration of disease, acupuncture intervention, control intervention, outcome measures, main results, and adverse events.

### Risk of bias assessment

Two reviewers (Jimin Park and Ji-Yeun Park) independently evaluated risk of bias for the included studies according to the Cochrane Collaboration’s risk of bias assessment tool [16]. The evaluated items for risk of bias were as follows.

(1) Was the method of randomization sequence generation adequate?

(2) Was the treatment allocation adequately concealed?

(3) Was the patient blinded to the intervention?

(4) Was the outcome assessor blinded to the intervention?

(5) Were incomplete outcome data adequately addressed?

(6) Are reports of the study free of suggestion of selective outcome reporting?

The reviewers rated the risk of bias for each item using ‘Yes’, ‘Unclear’, or ‘No’; ‘Yes’ meant a low risk of bias, ‘Unclear’ meant uncertain or unknown risk of bias, and ‘No’ meant a high risk of bias. Disagreements were resolved by discussion between the reviewers.

### Statistical analysis

Review Manager software (version 5.1 for Windows; The Nordic Cochrane Centre, Copenhagen, Denmark) was used for statistical analysis. Studies were classified and combined in the main analysis according to whether acupuncture was an alternative or an add-on treatment. Data were pooled using a random effects model. The impact of acupuncture on dichotomous data was expressed as the risk ratio (RR) of global symptoms persisting with the acupuncture intervention compared with the control, with 95% confidence intervals (CIs) (i.e. the RR of non-response). To define non-response, patient-reported global symptoms in ordinal assessments were divided into two groups (e.g. ‘poor’ or ‘good’ as non-response vs. ‘very good’ or ‘excellent’ as response). If different strata were used to define improvement, the cut-off point with the least improvement was taken (e.g. if the ordinal assessment was poor, good, or excellent, we utilized a poor vs. good or excellent comparison). In summary, a RR value of less than 1 indicated a lower risk of symptoms persisting or getting worse with acupuncture compared with the control. For continuous outcomes, the mean difference (MD) with a 95% CI was calculated.

Visual inspection of forest plots and a chi-square test with a significance level of *p* < 0.1 were used to assess heterogeneity among the included studies. To quantify inconsistencies among the studies, the *I*^2^ test was used with a value of 50% or more considered to indicate a substantial level of heterogeneity [17]. Subgroup analyses were conducted in terms of acupuncture intervention (e.g. manual acupuncture, electroacupuncture), grade of ankle sprain, and control type (e.g. usual care, sham acupuncture). Sensitivity analyses were also planned by including only those studies with low risk of bias or studies with sample size ≥ 40 per arm, and by varying the grouping of outcome measures. We analyzed trials with a low risk of bias for randomization and/or allocation concealment only [18,19] and investigated whether the intervention effect was affected. Studies with ≥ 40 participants per arm were analyzed separately to see whether any difference in intervention effect emerged [20]. For outcome measures, because it is common for Chinese trials to report outcomes based on an ordinal assessment (e.g. ‘excellent’, ‘very good’, ‘good’, ‘poor’), we also performed a sensitivity analysis by reanalyzing the dichotomous outcomes; we compared the ‘excellent, very good vs. good, poor’ scenario from our original analysis with an ‘excellent, (very) good vs. poor’ scenario to ascertain any discrepancies.

## Results

### Description of studies

**Figure 1:**
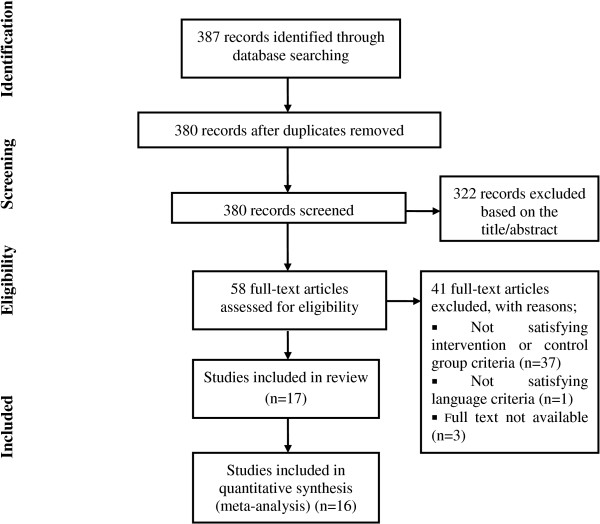
Flow diagram of literature search.

Our search terms yielded 387 records: five in the Cochrane Central Register of Controlled Trials, 21 in EMBASE, 42 in the CINAHL, 10 in SPORTDiscus, 10 in the AMED, two in Rehabilitation and Sports Medicine Source, 175 in the CNKI, 90 in PubMed, and 32 in the relevant Korean journals. After duplicate studies had been removed, 380 records were screened. Based on the title and abstract, 322 records were excluded; 162 articles were not specific to the topic of the review and 160 were not clinical studies or were non-randomized trials. Of the remaining 58, further studies were excluded as follows. (1) Thirty-seven studies did not satisfy the inclusion criteria for acupuncture or the control intervention: these were studies that compared different acupuncture styles (*n* = 9); studies of acupuncture vs. Chinese herbal medicine or bee venom, the efficacy of which has not been established (*n* = 8); trials where acupuncture was given with other therapies so that the effect of acupuncture *per se* could not be isolated (*n* = 9); and trials comparing the effect of another therapy given with acupuncture with that of acupuncture alone (*n* = 11). (2) One study published in French was excluded, and (3) we failed to obtain the full texts of three studies. Finally, 17 studies were included in our review and 16 studies reporting patient-reported global assessment outcome were pooled in the main analysis. Figure 1 shows a flow diagram of the literature search as recommended in Preferred Reporting Items for Systematic Reviews and Meta-Analyses (PRISMA) [21].

### Characteristics of the included studies

Details of the included studies are summarized in Tables 1 and 2 and Additional file 1.

**Table 1:** Summary of efficacy outcomes

**Author (year)**	**Treatment (no. of participants analyzed/randomized)**	**Outcome measures**	**Results**
**Acupuncture as an add-on treatment**			
Sun (2011) [24]	(A) MA + functional exercise (41/41)	1) PRGA^*^ at 14 d	1) NS
	(B) Functional exercise (41/41)	2) Time to cure (d)	2) (A) significantly better than (B)
Zheng (2010) [25]	(A) MA + PRICE (≤ 24 h), MA + EA (≥ 24 h) (40/40^†^; 27/40^‡^)	1) PRGA^* ^at 15 d	1) (A) significantly better than (B)
	(B) PRICE (≤ 24 h), EA (≥ 24 h) (33/33^†^; 12/33^‡^)	2) Time to cure	2) NS
Wei (2010) [37]	(A) WA + massage (30/30)	PRGA^§^ at 10 d	NS
	(B) TENS + massage (30/30)		
Tang (2010) [38]	(A) EA + massage + IR (30/30)	1) PRGA ^* ^at 10 d	1) (A) significantly better than (B)
	(B) Massage + IR (30/30^†^; 25/30^‡^)	2) Recurrence rate at 6 month follow-up (%)	2) NS
He (2010) [36]	(A) WA + small needle-knife therapy + drug injection + rehabilitation (87/87)	1) Pain (VAS) at immediately and two yrs after treatment	1) (A) significantly better than (B)
			2) (A) significantly better than (B)
	(B) Small needle-knife therapy + drug injection + rehabilitation (87/87)		
		2) QOL (SF-36) at immediately and two yrs after treatment	
	(C) WA + rehabilitation (87/87)		
He (2006) [29]	(A) MA + PRICE (≤ 24 h), EA + WA (≥ 24 h) (46/46^†^; 31/46^‡^)	1) PRGA^* ^at 15 d	1) (A) significantly better than (B)
	(B) PRICE (≤ 24 h), EA (≥ 24 h) (33/33^†^; 12/33^‡^)	2) Time to cure	2) NS
Li (2002) [31]	(A) MA + oral/topical HM (23/23)	PRGA^*^ at 8 d	(A) significantly better than (B)
	(B) Oral/topical HM (23/23)		
Ge (2000) [32]	(A) MA + oral HM (50/50)	PRGA^§^ at 10 d	NS
	(B) Oral HM (30/30)		
Yu (1999) [33]	(A) MA + topical NSAIDs (50/50)	PRGA^††^ at 7 d	NS
	(B) Topical NSAIDs (50/50)		
	(C) MA (50/50)		
Yu (2) (1999) [34]	(A) MA + topical NSAIDs + ice pack (30/30)	PRGA^††^ at 7 d	(A) significantly better than (B), (C), or (D)
	(B) Topical NSAIDs + ice pack (30/30)		
	(C) Ice pack (30/30)		
	(D) MA (30/30)		
Yu (1996) [35]	(A) MA + topical HM + ice pack (30/30)	PRGA^§^ at 7 d	(A) significantly better than (B)
	(B) Topical HM + ice pack (30/30)		
	(C) Ice pack (30/30)		
	(D) MA (30/30)		
Ruan (1995) [40]	(A) MA + massage (116/116)	PRGA^‡‡^	NS
	(B) MA (112/112)		
	(C) Massage (110/110)		
**Acupuncture alone vs. other therapy**			
Ni (2010) [26]	(A) MA (64/64^†^; 61/64^‡^)	1) PRGA^* ^at 3 d	1) (A) significantly better than (B)
	(B) Ice pack (≤ 24 h), hot pack (≥ 24 h) + oral HM + IR (59/59^†^; 45/59^‡^)	2) Time to cure	2) NS
Luo (2009) [27]	(A) EA (23/23)	PRGA^*^ at 4 wks	NS
	(B) Topical NSAIDs (23/23)		
Zhou (2008) [28]	(A) WA (26/26)	PRGA^**^ at 5 d	(A) significantly better than (B)
	(B) IR (23/23)		
Zhao (2005) [30]	(A) EA (43/43)	PRGA^*^ at 4 wks	NS
	(B) Oral/topical NSAIDs + hot pack (33/33)		
Wang (2005) [39]	(A) EA (27/27)	PRGA^*^ at 5 d	(A) significantly better than (B)
	(B) IR (30/30)		

*, cured/significantly improved/improved/failed; †, for outcome measure 1); ‡, for outcome measure 2); §, cured/improved/failed.

**, decrease rate ≥ 5/decrease rate ≤ 5 (mean score of pain and swelling).

††, significantly improved/improved/failed.

‡‡, cured/significantly improved/improved.

There were 4 trials which reported acupuncture alone vs. other treatment and acupuncture plus other treatment vs. other treatment [33-35,40]. They were put as acupuncture as an add-on treatment trials in this table.

d indicates days; EA, electroacupuncture; h, hours; HM, herbal medicine; IR, infrared radiation; MA, manual acupuncture; no., number; NS, no significant difference between groups; NSAIDs, non-steroidal anti-inflammatory drugs; PRGA, patient-reported global assessment; PRICE, protection, rest, ice, compression and elevation; QOL, quality of life; TENS, transcutaneous electrical nerve stimulation; VAS, visual analog scale; WA, warm acupuncture; wks, weeks; yrs, years.

**Table 2:** Summarized acupuncture interventions in the included studies

**Author (year)**	Acupuncture method (Fixed/partially individualized/individualized)*	**Treatment rationale**	**Regimen**	Acupuncture points**	**Response sought**	**Co-interventions**
Sun (2011)[24]	MA, fixed	Modern acupuncture (hand acupuncture)	14 sessions (once daily for 14 d)	Ex-UE205	De-qi***	Functional exercise
Zheng (2010)[25]	MA, fixed	Clinical experience	15 sessions (once daily for 5 d X 3)	LI15	De-qi	PRICE + EA
He (2010)[36]	WA, individualized	TCM theory	n.r	Tender points	De-qi	Small needle-knife therapy + drug injection + rehabilitation
Wei (2010)[37]	WA, individualized	TCM theory	10 sessions (once daily for 10 d)	Selected points from ST36, KI3, BL60, GB40, GB39, ST41, LR3 etc.	De-qi	Massage
Ni (2010)[26]	MA, partially individualized	TCM theory	3 sessions (once daily for 3 d)	Ex-UE140 + additional points (pain sensitive points on the contralateral wrist joint)	De-qi	None
Tang (2010)[38]	EA, partially individualized	TCM theory	10 sessions (once daily for 5 d X 2)	Ashi points(GB40, BL60, BL62, KI6) + additional points(ST41, GB39, GB34, ST36)	De-qi	Massage + IR
Luo (2009)[27]	EA partially individualized	TCM theory	12 sessions (six times per 2 wks X 2)	ST41, BL60, GB40 + ashi points	De-qi	None
Zhou (2008)[28]	WA, individualized	TCM theory	5 sessions (once daily for 5 d)	Tender points	n.r.	None
He (2006)[29]	MA + WA, fixed	TCM theory clinical experience	15 sessions (once daily for 5 d X 3)	MA, WA : GB34 EA : GB39, GB40, ST41, BL60, BL62, GB43	De-qi	PRICE + EA
Zhao (2005)[30]	EA, fixed	TCM theory	14 sessions (once per 2 days for 2 wks X 2)	Penetrating needling (GB40 and KI6)	De-qi	None
Wang (2005)[39]	EA, partially individualized	Modern experimental study	5 sessions (once daily for 5 d)	ST41, GB40, BL62, BL60, GB39, ashi points	De-qi	None
Li (2002)[31]	MA, partially individualized	n.r.	8 sessions (once daily for 8 d)	ST36, GB39, BL60, additional points (pain sensitive points on the contralateral Triple energizer meridian of wrist)	De-qi	Oral & topical HM
Ge (2000)[32]	MA, individualized	n.r	10 sessions	n.r	n.r	oral HM
Yu (1999)[33]	MA, fixed	TCM Theory	14 sessions (twice daily for 7 d)	ST36, GB39, KI3, BL60	n.r	Topical NSAIDs
Yu (1999)[34]	MA, fixed	n.r	14 sessions (twice daily for 7 d)	ST36, GB39, KI3, BL60	n.r	Topical NSAIDs + ice pack
Yu (1996)[35]	MA, fixed	n.r	7 sessions (once daily for 7 d)	ST36, GB39, KI3, BL60	n.r	Topical HM + ice pack
Ruan (1995)[40]	MA, partially individualized	n.r	Once daily	Ex-LE8, Ex-LE9, BL62, GB39, GB40, BL60, KI6, SP6, KI2, ST41, ST36, GB34, SP9, ashi points	n.r	Massage

*, Acupuncture method was classified into three categories based on the levels of individualization: ‘fixed’ means all patients receive the same treatment at all sessions, ‘partially individualized’ means using a fixed set of points to be combined with a set of points to be used flexibly, and ‘individualized’ means each patient receives a unique and evolving diagnosis and treatment [15]; **, Acupuncture point LI5 refers to 5th point of large intestine meridian and extra points have different nomenclature (e.g., Ex-UE3 means 3rd extra point in upper extremity). Ashi points mean local pain points; ***, De-qi means acupuncture-evoked specific sensations such as soreness, numbness, heaviness, and distention at the site of needle placement and these sensations may spread to other parts of the body.

d indicates days; EA, electroacupuncture; HM, herbal medicine; IR, infrared radiation; MA, manual acupuncture; n.r., not reported; NSAIDs, non-steroidal anti-inflammatory drugs; PRICE, protection, rest, ice, compression and elevation; TCM, traditional Chinese medicine; WA, warm acupuncture; wks, weeks.

#### Participants

Seventeen studies involving 1820 participants were included in our review. All were conducted in China and were published in Chinese. When divided into acute, chronic, and mixed ankle sprains based on a cut-off point of 6 months after onset [22,23], 12 studies [24-35] involved participants with acute ankle sprain, three studies [36-38] involved mixed participants, no study involved participants with chronic ankle sprain only, and one study [39] did not report disease duration but it was assumed that the participants had acute ankle sprain. Another study [40] mentioned ‘acute’ in the title, but 326 participants had had sprained ankles for less than 2 days and the other 12 for more than 3 days. Regarding the severity of the sprain, only four trials clearly reported that the participants had grade I or II injuries [24,28,31,39].

#### Acupuncture intervention

Highly variable acupuncture interventions were given either alone or as an add-on to the control intervention. Of the 17 included studies, nine [24-26,31-35,40] tested manual acupuncture, four [27,30,38,39] used electroacupuncture, three [28,36,37] used warm acupuncture, and one [29] used warm acupuncture in addition to manual acupuncture. Of five trials that assessed the effect of acupuncture alone, three studies evaluated electroacupuncture [27,30,39]; one each evaluated warm acupuncture [28] and manual acupuncture [26]. Fixed (i.e. all participants received the same treatment), partially individualized (using a fixed set of points with a further set of points to be used flexibly), and individualized (each participant received tailored treatment) acupuncture treatments were given; of the 17 studies, seven used fixed [24,25,29,30,33-35], six used partially individualized [26,27,31,38-40], and four used individualized treatments [28,32,36,37]. The number of acupuncture sessions ranged from three to 15 over 3 days to 4 weeks. De-qi – acupuncture-evoked specific sensations such as numbness, heaviness, soreness, or distention – was sought in 11 studies [24-27,29-31,36-39]. Fourteen studies [24-27,29-31,33-35,37-40] used 12 meridian points and/or extra points; two studies [28,36] used tender points, and one study [32] did not report the acupuncture points used. Details of acupuncture interventions are summarized in Table 2 based on the revised STRICTA [15].

#### Control intervention

A range of control interventions were used, including ice pack, exercise, bandage, analgesic drugs, herbal medicine, infrared radiation, tuina massage, hot pack, and TENS. No study adopted sham acupuncture as a control treatment (Table 1). More than one comparison group was used in five trials [33-36,40]. In nine trials evaluating the effect of acupuncture alone, usual care [26,27,30,33-35] or infrared radiation [26,28,39] was used as a control. Two studies adopted hot pack [26,30] or herbal medicine [26,35] as a control treatment. Only one study [40] used tuina massage. Of the other 12 trials testing acupuncture as an add-on treatment, seven [24,25,29,33-36] employed usual care and three used herbal medicine [31,32,35] or tuina massage [37,38,40]. One each adopted infrared radiation [38] and TENS [37].

#### Outcome measures

Outcome measures reported in the included studies were patient-reported global assessment (16 trials) [24-27,29-35,37-40], pain (one trial) [36], time to return to pre-injury level of work or sports (four trials) [24-26,29], recurrence rate (one trial) [38], health-related quality of life (one trial) [36], and adverse events (two trials) [34,35]. For patient-reported global assessment outcomes, a range of definitions was used to assess efficacy. We thus used the proportion of participants who had poor or good improvement (i.e. non-responders) in the main analysis.

### Risk of bias in the included studies

Most of the included trials were assessed as having a high risk of bias. Just three of the 17 studies reported an adequate method of sequence generation such as using a random number table or coin tossing [24,31,36]; among these, group assignment was adequately concealed using sealed opaque envelopes in only one trial [24]. For participant and personnel blinding, no trial was rated as having a low risk of bias because none of the trials was sham-controlled. Outcome measures were assessed by non-blinded participants in three studies [24,27,33]; the other 14 studies were rated as having an unclear risk of bias because we could not completely exclude the possibility that a blinded third party assessor might have evaluated global symptom improvement. All but one trial were assessed as having a low risk of bias because of having no missing outcome data; one study [24] did not report the number of participants analyzed in the outcome measure. Regarding selective outcome reporting, we could not locate and compare the protocols of any of the included studies; hence, we judged the risk of bias based on the described methods in each study. Six studies had a high risk of bias for selective outcome reporting because they were reporting unplanned outcome measurements [24-26,29,37,38] (Table 3).

**Table 3:** Risk of bias assessment*

	**Sun (2011)**[24]	**Zheng (2010)**[25]	**He (2010)**[36]	**Wei (2010)**[37]	**Ni (2010)**[26]	**Tang (2010)**[38]	**Luo (2009)**[27]	**Zhou (2008)**[28]	**He (2006)**[29]	**Zhao (2005)**[30]	**Wang (2005)**[39]	**Li (2002)**[31]	**Ge (2000)**[32]	**Yu (1999)**[33]	**Yu (1999)**[34]	**Yu (1996)**[35]	**Ruan (1995)**[40]
1. Was the method of randomization adequate?	Y	U	Y	U	U	U	U	U	U	U	U	Y	U	U	U	U	U
2. Was the treatment allocation concealed?	Y	U	U	U	U	N	N	U	U	N	U	U	U	U	U	U	U
3. Was the patient blinded to the intervention?	N	N	N	N	N	N	N	N	N	N	N	N	N	N	N	N	N
4. Was the outcome assessor blinded to the intervention?	U	U	N	U	U	U	N	U	U	N	U	U	U	U	U	U	U
5. Were incomplete outcome data adequately addressed?	N	Y	Y	Y	Y	Y	Y	Y	Y	Y	Y	Y	Y	Y	Y	Y	Y
6. Are reports of the study free of suggestion of selective outcome reporting?	N	N	Y	N	N	N	Y	Y	N	Y	Y	Y	Y	Y	Y	Y	Y

*, Based on the risk of bias assessment tool from the Cochrane Handbook for Systematic Reviews of Interventions [16]; ‘Y’ indicates “Yes (low risk of bias)”; ‘U’, “Unclear”; ‘N’, “No (high risk of bias)”; A study with a low risk of bias was defined as a study receiving ‘Y’ for randomization and/or allocation concealment.

### Effects of acupuncture on primary outcome

**Figure 2:**
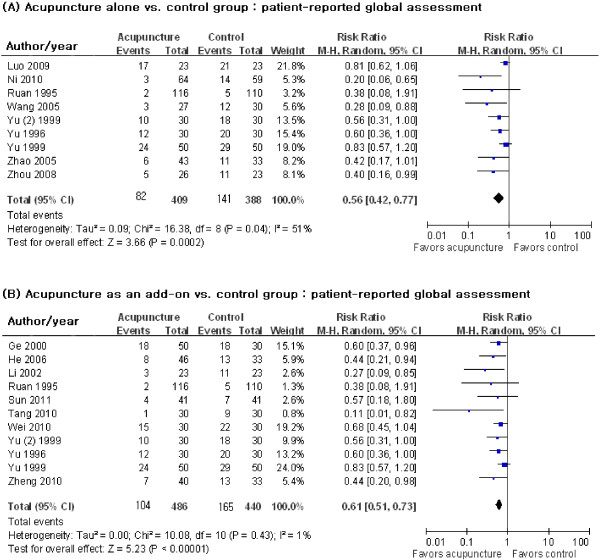
Effects of acupuncture for ankle sprains according to the acupuncture as an alternative or add-on treatment.

The key outcomes from the included studies are provided in Table 1 and Figure 2. We evaluated primary outcomes of patient-reported global assessment. Pain was considered if a global assessment was not available.

#### Effects of acupuncture as an alternative treatment

Nine trials with 797 participants [26-28,30,33-35,39,40] reported global symptom improvement as a dichotomous outcome. Approximately nine sessions of acupuncture were given over 11.5 days. Acupuncture had a statistically significant effect in reducing the global symptoms of ankle sprain (RR of symptoms persisting with acupuncture = 0.56; 95% CI 0.42–0.77; Figure 2 (A)). However, there was substantial heterogeneity among the trials (*χ*^2^ = 16.38, degrees of freedom (df) = 8, *p* = 0.04, *I*^2^ = 51%).

#### Effects of acupuncture as an add-on treatment

Eleven trials with 926 participants reported the add-on effect of acupuncture [24,25,29,31-35,37,38,40]. A median of 10 sessions of acupuncture over nine days was provided. When added to other treatment, acupuncture statistically significantly improved global symptoms compared with the other treatment only (RR of symptoms persisting with acupuncture = 0.61; 95% CI 0.51–0.73; Figure 2 (B)). There was no significant heterogeneity among the studies (*χ*^2^ = 10.08, df = 10, *p* = 0.43, *I*^2^ = 1%).

#### Effects of acupuncture on pain intensity

One study reported pain intensity on a VAS both immediately and more than 2 years after treatment [36]. Immediately after treatment, warm needling significantly alleviated pain compared with the control group (1.32 ± 0.42 vs. 6.55 ± 1.76, MD –5.23, 95% CI –5.61 to –4.85). At long-term follow-up of 28.8 months on average, the analgesic effect was maintained (1.01 ± 0.15 vs. 5.89 ± 1.93, MD –4.88, 95% CI –5.29 to –4.47).

### Effects of acupuncture on secondary outcomes

#### Time to achieve pre-injury level of work or sports

Four studies reported time to cure [24-26,29]. Of these, one study [24] reported that acupuncture in addition to functional exercise shortened the time to return to normal activity by 3.4 days compared with a functional exercise only group (5.2 ± 0.7 vs. 8.6 ± 1.4, MD –3.40, 95% CI –3.88 to –2.92). In the other three studies [25,26,29], participants were no more likely to have recovered within 1 week than were those in the control group, regardless of whether they were given acupuncture as an add-on (two trials, RR 2.49, 95% CI 0.60 to 10.29, *I*^2^ = 0%) or an alternative treatment (one trial, RR 1.21, 95% CI 0.99–1.47).

#### Ankle instability and swelling

No included study reported on ankle instability and/or swelling as a separate outcome measure; most of the studies reported a composite measure of patient-reported global symptoms.

#### Recurrence of ankle sprain

One study [38] reported that one participant in the acupuncture group and five in the control group had suffered a re-injury at 6-month follow-up (RR 0.17, 95% CI 0.02–1.33).

#### Health-related quality of life

One study [36] reported quality of life using the SF-36 immediately and more than 2 years after treatment. Immediately after treatment, the acupuncture group reported significantly better quality of life than the control group (91.25 ± 10.16 vs. 76.53 ± 5.24, MD 14.72, 95% CI 12.32–17.12). At 2-year follow up, the effect remained significant (93.62 ± 9.05 vs. 62.31 ± 6.67, MD 31.31, 95% CI 28.95–33.67).

#### Adverse events

Two studies [34,35] reported mild adverse events such as a mild allergic response to medication, from which the patients (three participants) recovered after the drug was stopped.

### Subgroup analyses

We conducted subgroup analyses based on the following predefined characteristics: type of acupuncture intervention, grade of ankle sprain, and control type.

#### Acupuncture type

Manual acupuncture [24,25,31-35,40] had an additional effect on symptom improvement compared with controls (eight trials, RR 0.62, 95% CI 0.50–0.77, *I*^2^ = 0%). When manual acupuncture was given as an alternative [26], the RR of symptoms persisting with acupuncture was 0.20 (95% CI 0.06–0.65). Electroacupuncture as a sole treatment [27,30,39] had no significant benefit compared with oral/topical NSAIDs or infrared radiation (three trials, RR 0.50, 95% CI 0.20–1.22, *I*^2^ = 76%). When added to massage and infrared radiation [38], the effect of electroacupuncture was statistically significantly better than that of massage and infrared radiation only (one trial, RR 0.11, 95% CI 0.01–0.82).

#### Grade of ankle sprain

There were insufficient data for subgroup analysis of sprain severity. Only four trials [24,28,31,39] clearly reported that their participants had grade I or II injuries; the RR of symptoms persisting with acupuncture was 0.39 (two trials, 95% CI 0.18–0.88, *I*^2^ = 0%) when acupuncture was given as an add-on treatment and 0.35 (two trials, 95% CI 0.17–0.71, *I*^2^ = 0%) when it was an alternative treatment.

#### Control type

1. Acupuncture vs. oral/topical NSAIDs

(1) Acupuncture vs. NSAIDs

**Figure 3:**
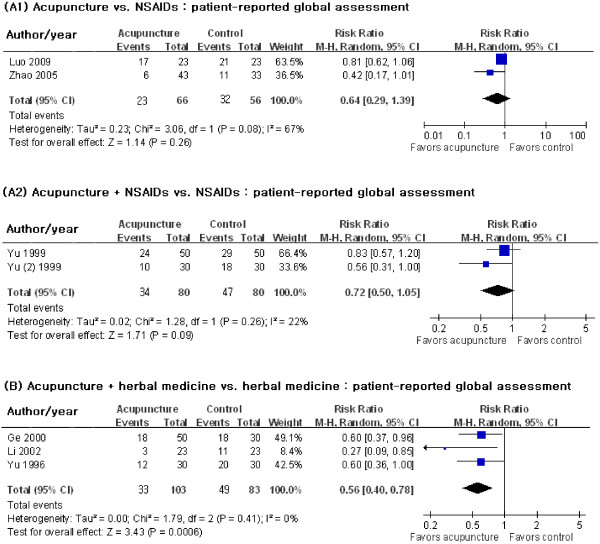
Effects of acupuncture for ankle sprains according to the control groups.

Two trials with 122 participants tested the effect of acupuncture on global symptom improvement against oral/topical NSAIDs [27,30]. There was no statistically significant difference between the groups (Figure 3 (A1); RR 0.64, 95% CI 0.29–1.39, *I*^2^ = 67%).

(2) Acupuncture plus NSAIDs vs. NSAIDs

Acupuncture had no additional effect on global symptom improvement compared with oral/topical NSAIDs only [33,34] (Figure 3 (A2); RR 0.72, 95% CI 0.50–1.05, *I*^2^ = 22%).

2. Acupuncture plus herbal medicine vs. herbal medicine

Symptoms persisted in significantly fewer participants when acupuncture was added to oral/topical herbal medicine compared with the medicine alone [31,32,35] (Figure 3 (B); RR 0.56, 95% CI 0.40–0.78, *I*^2^ = 0%).

### Sensitivity analyses

We performed sensitivity analyses by excluding studies with predefined less desirable characteristics, as follows.

#### Risk of bias

When the analysis was limited to two studies with a low risk of bias for random sequence generation and/or allocation concealment [24,31], the add-on effect of acupuncture on patient-reported global assessment remained significant (RR 0.39, 95% CI 0.18–0.88, *I*^2^ = 0%).

#### Sample size

When four studies with ≥ 40 participants per group [24,26,33,40] were pooled, there was no significant difference in the risk of symptoms persisting or worsening between the acupuncture and control groups (RR 0.50, 95% CI 0.24–1.05, *I*^2^ = 55%).

#### Outcome measures

For 16 studies reporting a dichotomous outcome based on an ordinal assessment, we compared the ‘excellent, very good’ vs. ‘good, poor’ scenario (16 trials, RR 0.55, 95% CI 0.45–0.69, *I*^2^ = 40%) with an ‘excellent, (very) good vs. poor’ scenario (RR 0.26, 95% CI 0.18–0.38, *I*^2^ =0%); the difference remained significant without variability.

## Discussion

### Summary of evidence

This systematic review aimed to assess the evidence in support of acupuncture treatment for ankle sprains. Seventeen RCTs were included that investigated the effect on global symptom improvement of acupuncture as an alternative or an add-on to other treatment. In the evaluation of acupuncture compared with other treatments, acupuncture had a therapeutic benefit in improving global symptoms (RR of symptoms persisting with acupuncture = 0.56, 95% CI 0.42–0.77). However, this is probably an overestimate due to the heterogeneity (*I*^2^ = 51%) and high risk of bias of the included studies.

Compared with other treatments alone, acupuncture as an adjunct significantly alleviated the global symptoms of ankle sprain without significant variability (RR of symptoms persisting with acupuncture = 0.61, 95% CI 0.51–0.73).

A sensitivity analysis of the trials with a low risk for selection bias suggested that the beneficial effect of acupuncture was maintained. The effect of acupuncture was no more significant when the analysis was limited to studies with adequate sample size. Acupuncture was more effective than various controls in relieving pain, facilitating return to normal activity, and promoting quality of life, but these analyses were based on only a small number of studies. Acupuncture did not appear to be associated with serious adverse events, but the evidence is limited.

### Risk of bias

Most of the included studies suffered from a serious risk of bias. Only three studies had a low risk of bias for adequate randomization and/or allocation concealment. It is well known that inadequate allocation concealment/random sequence generation leads to overestimation of treatment effects [18,19]. Although the included studies uniformly reported no difference in baseline characteristics between groups, we cannot exclude the possibility that selection bias may have played a role. However, when we limited our main analysis to those studies rated as having a low risk of bias for randomization/allocation concealment, the benefit of acupuncture remained significant.

Because of the small number of studies, we could not formally test for funnel plot asymmetry to detect small-study effects (a tendency for the intervention effects detected in smaller studies to differ from those in larger studies [41]). Chinese studies may have been more likely to publish positive outcomes [42,43], but more importantly, the effect size of small studies in this review may have been inflated because of poor methodological design and conduct [44]. Because it is well known that small, poor-quality studies tend to spuriously inflate the effect of an intervention, we need to be more conservative in the interpretation of such results.

### Limitations of this review

Although we made every endeavor to find all relevant trials in a range of databases and related journals, comprehensive searches do not necessarily remove publication bias or language bias. All trials were conducted in China and published in Chinese journals in the Chinese language. Egger *et al.*[43] reported that inclusion of studies published in non-English languages or in journals that are not indexed in Medline is likely to increase the degree of funnel plot asymmetry in systematic reviews, and this may be relevant to the present review.

The included trials were mostly of poor quality; thus, the reported data are likely to be overestimated. In addition, the small sample size of the studies may have resulted in heterogeneity of the effect size. Moore *et al.*[20] reported in a simulation study that at least 40 participants per arm are required to obtain clinically relevant results in trials relating to pain. Our sensitivity analysis based on sample size (i.e. trials with ≥ 40 per group only [24,26,33,36]) found no significant benefit from acupuncture.

Finally, as is usual in Chinese acupuncture trials, most of the studies in our review used various subjective outcomes. Because no study compared acupuncture with sham acupuncture, this makes outcome assessment blinding even more crucial. Failure to blind outcome assessment may have influenced the studies’ results.

### Implications for practice

This systematic review suggests that there is insufficient high-quality evidence supporting the use of acupuncture as an alternative treatment to improve global symptoms of ankle sprain. Acupuncture provided a significant benefit as an add-on treatment and a sensitivity analysis of high-quality trials supports this finding, but the number of studies is too small to strongly recommend this use.

There are several issues worth considering before we make any judgments on the use of acupuncture for ankle sprain in practice. First, the tested acupuncture interventions varied widely across the trials in terms of type, points, number of sessions, and duration of treatment. Thus, it is difficult to determine whether these interventions were truly adequate or optimal.

Second, the control interventions used in the included studies also varied; they included usual care (e.g. ice pack, exercise, bandage, analgesics), herbal medicine, infrared radiation, tuina massage, hot pack, and TENS. Studies on the efficacy of topical NSAIDs compared with oral NSAIDs in the treatment of acute pain were conducted recently due to the adverse effects of oral NSAIDs such as gastrointestinal complications and cardiovascular toxicity. In a review of their use for musculoskeletal pain [45], topical NSAIDs were found to have comparable efficacy and better safety compared with oral NSAIDs when used for acute pain including sprains and strains, and the effect of topical NSAIDs was 1.6 times better than that of placebo at 7 days. In the present review, acupuncture as an add-on or alternative treatment had no better effect than oral/topical NSAIDs but was associated with few side effects. Acupuncture was significantly effective for symptom improvement only when added to oral/topical herbal medicine.

Third, the outcome measures of the included studies were not consistent. The clinical relevance of any benefit of acupuncture is not obvious [46], and because there are no reliable data on the minimal clinically important difference relating to patient-reported global symptom improvement in ankle sprain, we may only infer that the effect of acupuncture is small [46].

Fourth, all of the trials included in this review were conducted in China. Acupuncture may be highly culture specific and further research is necessary to investigate whether the interventions reported here are applicable to and acceptable in other countries. Undergoing acupuncture every day may not be a feasible treatment schedule outside China.

Lastly, clinically meaningful information on the severity of injury and follow-up data were sparse in the included trials. Thus, the available evidence prevents us from determining whether the effects of acupuncture varied with the severity of injury or how long its benefit was maintained.

In summary, evidence supporting the use of acupuncture in patients with ankle sprain is currently inconclusive.

### Implications for research

To obtain a more definitive answer to the question of the efficacy of acupuncture for ankle sprain, we need more carefully designed and conducted trials. Researchers should use adequate randomization methods and ensure that group assignment is adequately concealed, because these are both critical in avoiding systematic differences between the baseline characteristics of the groups that are compared (i.e. selection bias). Because it is almost impossible for the therapist to be blinded to the acupuncture intervention provided, it may be more important to blind the participants and outcome assessor. There was no study with sham control in this review, so performance bias is likely to have played a part in our findings.

In the future, a sham-controlled trial is needed to avoid performance bias. To maintain outcome assessor blinding, validated assessment tools are required. Recent studies have tested the validity, reliability, and responsiveness of relevant scales [47,48]. For example, a study has tested the validity and responsiveness of the ankle functional score (AFS) based on fundamental functional outcomes such as pain, swelling, weight bearing, stability, and gait, and concluded that the AFS is easy to use and might be employed alongside subjective clinical assessment to evaluate recovery after acute ankle sprain [48]. Using not only subjective patient-reported symptom improvement, but also validated outcome measures, and ensuring outcome assessment blinding, should be considered for future trials.

## Conclusions

Given the methodological flaws of the included studies, the available evidence is insufficient to recommend acupuncture as an evidence-based treatment option for ankle sprain. Further well-designed and conducted trials are needed to draw a definitive conclusion.

## Competing interests

The authors declare that they have no competing interests.

## Authors’ contributions

HSL and JMP designed this review, searched databases, and screened trials for inclusion. JMP and JYP extracted data, evaluated studies and it was checked by HSL. HSL and JMP performed analyses and discussed with HJP and SKH. All authors read and approved the final manuscript.

## Pre-publication history

The pre-publication history for this paper can be accessed here:

http://www.biomedcentral.com/1472-6882/13/55/prepub

## Supplementary Material

Additional file 1 Characteristics of the included studies. Click here for file
