# Supplementary material for: Acupuncture for ankle sprain: systematic review and meta-analysis
Source: BMC Complement Altern Med. 2013 Mar 4;13:55. doi: 10.1186/1472-6882-13-55 (PMC3606608; doi:10.1186/1472-6882-13-55)
Supplement: Additional file 1 — Characteristics of the included studies. [file 1472-6882-13-55-S1.doc]

**Additional file 1.** Characteristics of the Included Studies

| **Author (year), country** | **Design** | **Participant** | **Duration of**  **disease** | **Excluded if fracture?** | **Acupuncture group (no. of participants analysed/**  **randomised)** | **Control group**  **(no. of participants analysed/**  **randomised)** | **Outcome measures : evaluation time** | **Main results** | **AE** |
| --- | --- | --- | --- | --- | --- | --- | --- | --- | --- |
| Sun (2011)  [21]  China | Parallel, 2 arms | 82 participants aged 21-30 yrs (range,  M/F= 62/20) | ≤ 6 h | Y | (A) MA + functional exercise (41/41) | (B) Functional exercise (41/41) : elastic bandage, restrict active and passive movements of ankle joint, assist exercise | 1) Patient-reported global assessment ^*^  2) Time to disappear pain (d)  : outcomes measured ^†^ | 1) (A) significantly better than (B) (P<0.05)  2) (A) significantly lower than (B) (P<0.05) | n.r. |
| Zheng  (2010)  [22]  China | Parallel, 2 arms | 73 participants aged 14-57 yrs (range,  M/F=40/33) | ≤ 21 d | Y | (A) Cotton pad pressure + bandage + ice pack + MA (ankle sprain ≤ 24h), EA + MA (ankle sprain ≥ 24h)  (40/40 ^‡ §^ ; 27/40 ^**^) | (B) Cotton pad pressure + bandage + ice pack (ankle sprain ≤ 24h) + EA (ankle sprain ≥ 24h) (33/33 ^‡ §^ ; 12/33 ^**^) | 1) Patient-reported global assessment ^*^  (cure rate)  2) Patient-reported global assessment ^*^ (efficacy rate)  3) Time to cure  : outcomes measured ^†^ | 1) (A) significantly better than (B) (P<0.05)  2) (A) significantly better than (B) (P<0.05)  3) (A) significantly lower than (B) (P<0.05) | n.r |
| He  (2010)  [33]  China | Parallel, 3 arms | 261 participants aged 51-68 yrs (range) | mean duration of 8 months | Y | (A) Improved silver needle acupuncture moxibustion + small needle-knife therapy + drug injection + ankle rehabilitation (87/87) | (B) Silver needle acupuncture moxibustion + ankle rehabilitation (87/87)  (C) Small needle-knife therapy + drug injection + ankle rehabilitatation (87/87) | 1) Pain  - VAS  2) Quality of life  - SF-36  : outcomes measured at before, immediately after and 28.75±9.26 months after the end of the sessions | 1) (A) significantly better than (C) (P<0.05)  (B) significantly better than (C) (P<0.05)  (A) significantly better than (B)  2) (A) significantly better than (C) (P<0.05)  (B) significantly better than (C) (P<0.05)  (A) significantly better than (B) (P<0.05) | n.r |
| Wei  (2010)  [34]  China | Parallel, 2 arms | 60 participants aged 24-58 yrs (range, M/F=41/19) | mean duration of 32 months (1-60 months) | Y | (A) Massage + WA (30/30) | (B) Massage + TENS (30/30) | 1) Patient-reported global assessment ^††^ (cure rate)  2) Patient-reported global assessment ^††^ (efficacy rate)  : outcomes measured ^†^ | 1) (A) significantly better than (B) (P<0.05)  2) NS | n.r. |
| Ni  (2010)  [23]  China | Parallel, 2 arms | 123 participants aged 13-63 yrs (range, M/F=53/70) | 1-5 d | Y | (A) MA  (64/64 ^‡^ ; 61/64 ^§^) | (B) Ice pack (duration ≤ 24h) + hot pack (duration ≥24h) + herbal medicine (oral) + IR (59/59 ^‡^ ; 45/59 ^§^) | 1) Patient-reported global assessment ^*^  2) Time to disappear pain (d)  : outcomes measured ^†^ | 1) (A) significantly better than (B) (P<0.05)  2) (A) significantly lower than (B) (P<0.05) | n.r. |
| Tang  (2010)  [35]  China | Parallel,  2 arms | 60 participants aged 18-23 yrs (range, M/F=38/22) | 2 months  -4 yrs | Y | (A) EA + massage + IR (30/30) | (B) Massage + IR  (30/30 ^‡^ ; 25/30 ^§^) | 1) Patient-reported global assessment ^*^  2) Recurrence rate ( 6 months after the sessions, %)  : outcomes measured at the end of the sessions and 6 months after the end of the sessions | 1) (A) significantly better than (B) (P<0.05)  2) (A) significantly lower than (B) (P<0.01) | n.r. |
| Luo  (2009)  [24]  China | Parallel, 2 arms | 46 participants aged 15-59 yrs (range, M/F=28/18) | 1-6 months | n.r. | (A) EA (23/23) | (B) Medicine (23/23):  topical NSAIDs (diclofenac) bid | 1) Patient-reported global assessment ^*^  : outcomes measured ^†^ | 1) (A) significantly better than (B) (P<0.05) | n.r. |
| Zhou  (2008)  [25]  China | Parallel, 2 arms | 49 participants aged 17-45 yrs (range, M/F=21/28) | 2-5 d | Y | (A) WA (26/26) | (B) IR (23/23) | 1)Patient-reported global assessment  : outcomes measured ^†^ | 1) (A) significantly better than (B) (χ^2^=4.54, P<0.05) | n.r. |
| He  (2006)  [26]  China | Parallel, 2 arms | 79 participants aged 16-70 yrs (range, M/F=28/51) | 2 h-15 d | Y | (A) Cotton pad pressure + bandage + ice pack + MA (ankle sprain ≤ 24h), EA + WA  (ankle sprain≥24h)  (46/46 ^‡ §^ ; 31/46 ^**^) | (B) Cotton pad pressure + bandage + ice pack (ankle sprain ≤ 24h), EA (ankle sprain≥24h)  (33/33 ^‡^ ^§^ ; 12/33 ^**^) | 1) Patient-reported global assessment ^*^  (cure rate)  2) Patient-reported global assessment ^*^  (efficacy rate)  3) No. of treatments required for cure  : outcomes measured ^†^ | 1) (A) significantly better than (B) (χ^2^=7.4581, P<0.01)  2) (A) significantly better than (B) (χ^2^=6.1526, P<0.05)  3) (A) significantly lower than (B) (χ^2^=7.4023, P<0.05) | n.r. |
| Zhao  (2005)  [27]  China | Parallel, 2 arms | 76 participants aged 16-53 yrs (range, M/F=43/33) | 1-6 months | n.r. | (A) EA (43/43) | (B) Medicine + hot pack (33/33):  oral NSAIDs (indometacin) 50mg, topical NSAIDs (diclofenac) bid | 1) Patient-reported global assessment ^*^  (cure rate)  2) Patient-reported global assessment ^*^  (efficacy rate)  : outcomes measured ^†^ | 1) (A) significantly better than (B) (χ^2^=7.86, P<0.05)  2) (A) significantly better than (B) (χ^2^=5.78, P<0.05) | n.r. |
| Wang  (2005)  [36]  China | Parallel, 2 arms | 57 participants aged 13-60 yrs (range, M/F=45/12) | n.r. (presumably acute) | Y | (A) EA (27/27) | (B) IR (30/30) | 1) Patient-reported global assessment ^*^  : outcomes measured ^†^ | 1) (A) significantly better than (B) (P<0.01) | n.r. |
| Li  (2002)  [28]  China | Parallel  2 arms | 46 participants | ≤ 2 wks | Y | (A) MA + herbal medicine (23/23) | (B) Herbal medicine (23/23) : oral and topical, twice daily/ once per 2 d for 8 d | 1) Patient-reported global assessment ^*^  (cure rate)  2) Patient-reported global assessment ^*^ (efficacy rate)  : outcomes measured ^†^ | 1) (A) significantly better than (B) (P<0.05)  2) (A) significantly better than (B) ( P<0.05) | n.r. |
| Ge  (2000)  [29]  China | Parallel  2 arms | 80 participants aged 15-25 yrs (range, M/F=55/25) | ≤ 3 d | n.r. | (A) MA + herbal medicine (50/50) | (B) Herbal medicine (30/30) : oral | 1) Patient-reported global assessment ^†^ : outcomes measured ^†^ | 1) (A) significantly better than (B) (χ^2^=8.808, P<0.05)) | n.r |
| Yu  (1999)  [30]  China | Parallel  3 arms | 150 participants aged 18-23 yrs (range, M/F=95/55) | ≤ 3 d | Y | (A) MA + medicine (50/50) | (B) Medicine (50/50)  : topical NSAIDs (ibuprofen)  (C) MA (50/50) | 1) Patient-reported global assessment ^‡‡^ : outcomes measured ^†^ | 1) (A) significantly better than (B) ( P<0.05)  (A) significantly better than (C) (P<0.01) | n.r |
| Yu  (1999)  [31]  China | Parallel  4 arms | 120 participants aged 18-23 yrs (range, M/F=70/50) | ≤ 3 d | Y | (A) MA + medicine + ice pack (30/30) | (B) Medicine + ice pack (30/30) : topical NSAIDs (ibuprofen)  (C) Ice pack (30/30)  (D) MA (30/30) | 1) Patient-reported global assessment ^‡‡^ : outcomes measured ^†^ | 1) (A) significantly better than (B) ( P<0.05)  (A) significantly better than (C) (P<0.01)  (A) significantly better than (D) (P<0.01) | mild drug allergy  (no.=1) |
| Yu  (1996)  [32]  China | Parallel  4 arms | 120 participants aged 16-22 yrs (range, M/F=64/56) | ≤ 3 d | Y | (A) MA + herbal medicine + ice pack (30/30) | (B) Herbal medicine + ice pack (30/30) : topical  (C) Ice pack (30/30)  (D) MA (30/30) | 1) Patient-reported global assessment ^††^ : outcomes measured ^†^ | 1) (A) significantly better than (B) ( P<0.05)  (A) significantly better than (C) (P<0.01)  (A) significantly better than (D) (P<0.01) | mild drug allergy  (no.=2) |
| Ruan  (1995)  [37]  China | Parallel  3 arms | 338 participants aged 15-52 yrs (range, M/F=252/86) | ≤ 2 d (no.=326)  ≥ 3 d  (no.=12) | n.r | (A) MA + massage (116/116) | (B) MA (112/112)  (C) Massage (110/110) | 1) Patient-reported global assessment ^§§^ (cure rate)  : outcomes measured ^†^ | 1) (A) significantly better than (B) (P<0.01)  (A) significantly better than (C) (P<0.01) | n.r |

*, cured/significantly improved/improved/failed

†, at the end of the sessions

‡, in case of outcome measure 1)

§, in case of outcome measure 2)

**, in case of outcome measure 3)

††, cured/improved/failed

‡‡, significantly improved/improved/failed

§§, cured/significantly improved/improved

Data are expressed as mean±SD unless stated otherwise.

AE indicates adverse events; d, days; EA, electroacupuncture; F, female; h, hours; IR, infrared radiation; M, male; MA, manual acupuncture; No, number; n.r., not reported; NS, no significant difference between groups; SF-36, short form 36 health survey scale; TENS, Transcutaneous Electrical Nerve Stimulation; VAS, visual analog scale; WA, warm acupuncture; wks, weeks; Y, yes; yrs, year.
